# Measuring the Contractile Response of Isolated Tissue Using an Image Sensor

**DOI:** 10.3390/s150409179

**Published:** 2015-04-20

**Authors:** David Díaz-Martín, José Gerardo Hernández-Jiménez, Manuel Rodríguez-Valido, Ricardo Borges

**Affiliations:** 1 Industrial Engineering Department, School of Physics, La Laguna University, Av. Astrofísico Francisco Sánchez s/n, San Cristóbal de La Laguna, 38200 Santa Cruz de Tenerife, Spain; E-Mail: mrvalido@ull.edu.es; 2 Bioorganic University Institute “Antonio González”, La Laguna University, Av. Astrofísico Francisco Sánchez s/n, San Cristóbal de La Laguna, 38200 Santa Cruz de Tenerife, Spain; E-Mails: jghdez@gmail.com (J.G.H.-J.); rborges@ull.es (R.B.); 3 Pharmacology Unit, School of Medicine, La Laguna University, Av. Astrofísico Francisco Sánchez s/n, San Cristóbal de La Laguna, 38200 Santa Cruz de Tenerife, Spain

**Keywords:** image sensor, isolated tissue, contractile response, photogrammetry, image-processing algorithm, monitoring drug effects

## Abstract

Isometric or isotonic transducers have traditionally been used to study the contractile/relaxation effects of drugs on isolated tissues. However, these mechanical sensors are expensive and delicate, and they are associated with certain disadvantages when performing experiments in the laboratory. In this paper, a method that uses an image sensor to measure the contractile effect of drugs on blood vessel rings and other luminal organs is presented. The new method is based on an image-processing algorithm, and it provides a fast, easy and non-expensive way to analyze the effects of such drugs. In our tests, we have obtained dose-response curves from rat aorta rings that are equivalent to those achieved with classical mechanic sensors.

## 1. Introduction

The quantification of contractile responses was one of the first methods described in pharmacology. As smooth muscle contraction/relaxation is a phenomenon that can be visualized directly, scientists studied the movements of the isolated intestine and other tissues, immersing the preparations in a tissue-bath filled with a saline buffer and recording them in kymographs using smoked paper. With minor variations, this technique is still use today, improvements coming from the incorporation of multichannel recordings due to the use of mechanical transducers, along with computer acquisition and analysis. The differences observed between isotonic and isometric/auxotonic recordings in tissues are traditionally studied using such methods. However, as vessels contract against their interior pressure and in order to avoid the tissue damage caused by the hooks or threads used in mechanical transducers, a system has been developed based on the recording of contractions by monitoring the inner pressure.

Arterial vessels are dynamic structures that are exposed to a pulsatile blood flow. Given that their muscle tone may be regulated by either innervation and/or circulating hormones, evidently none of the methods currently available to measure their contraction is perfect. Moreover, while a vast amount of direct data that can be obtained from organ baths, using the traditional approach to run assays on dozens of isolated tissue preparations is not commonly performed in pharmacology laboratories. There are a number of reasons for this, but the cost and the bench space required for such large equipment have been key deterrents.

In this paper, we describe a new method that allows the contractile effect of drugs to be measured on blood vessel rings using an image sensor (video-camera). Compared with traditional mechanical transducers, this method offers an easy, fast and non-expensive way to carry out studies into contractile responses using less complicated equipment.

## 2. Materials and Methods

**Figure 1:**
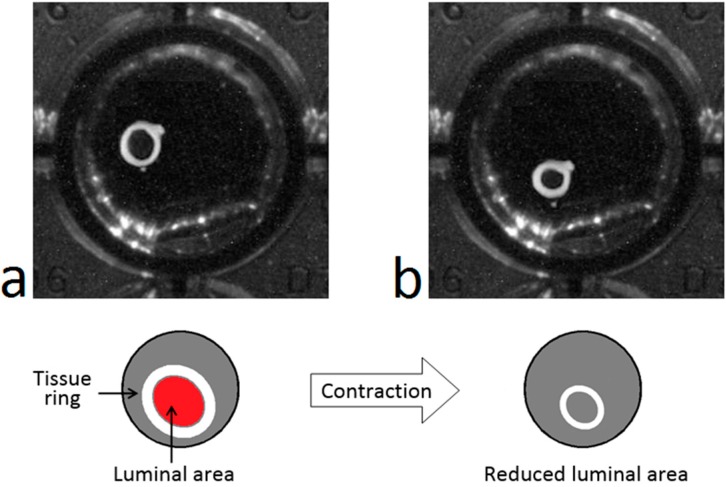
Representation of the contractile response: Rat aorta ring before (**a**) and after (**b**) the application of a contractile drug.

**Figure 2:**
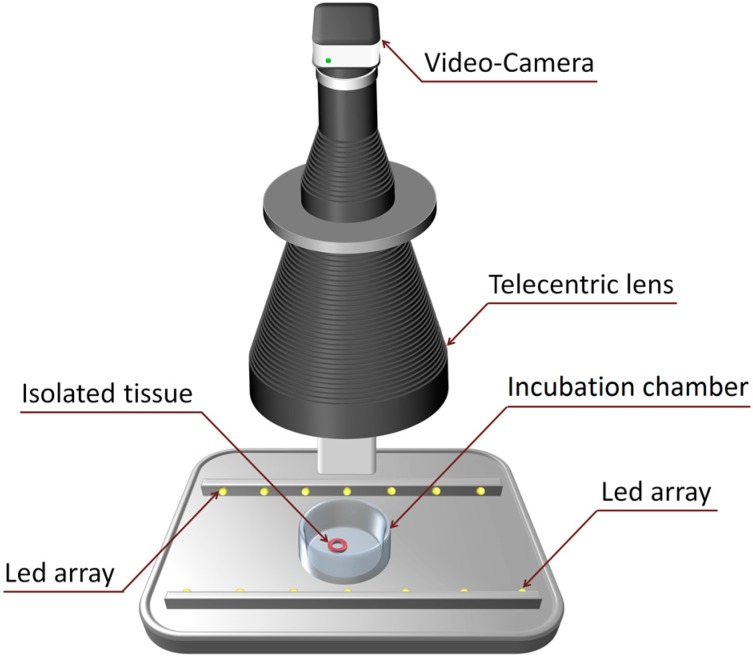
Image acquisition system.

When an artery ring contracts after the application of a drug, its lumen diameter decreases gradually (see Figure 1). This contractile response can be registered in a temporal sequence of images that can be acquired automatically with an image acquisition system such as that shown in Figure 2.

This system is comprised of a high resolution video-camera, a telecentric lens and a simple illumination system. To monitor the contractions, we have developed an image-processing algorithm that detects the tissue ring when a new image is captured and that measures its lumen area by calculating the number of pixels enclosed within the ring. At the same time, a specific software application calculates an on-line dose-response curve that can be readily converted into surface units.

### 2.1. Video-Camera

**Figure 3:**
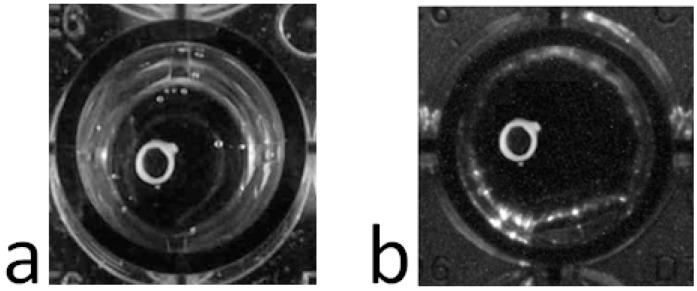
Image of the same aorta ring under conditions of equal light without (**a**) and with (**b**) a telecentric lens.

Any commercial USB controlled video-camera can be used with the acquisition software, such as a simple webcam. We use a UI1490LE camera (Ueye, IDS Imaging, Obersulm, Germany) the high resolution of which (10 Megapixels) allows us to record very mild contractions. The camera is situated ≈60 cm above the incubation chamber and we take advantage of the camera’s telecentric lens (OptoEngineering, Mantova, Italy) to eliminate image aberrations like reflections and geometric distortions, or those related to perspective (Figure 3). Even without a telecentric lens, the influence of many aberrations can be dampened by image processing.

### 2.2. Software Application

The software application is user-friendly with a simple graphical interface based on a windows environment and menus. The video camera can be adjusted via the application, which can also control image acquisition. In addition, the user can configure the experimental parameters and monitor the contractile response in real time. The results can be stored in a tabulated program (e.g., Excel) or as a text file. The application was designed on Microsoft Windows^®^ OS and implemented using MATLAB^®^ [1].

## 3. Image-Processing Algorithm

**Figure 4:**
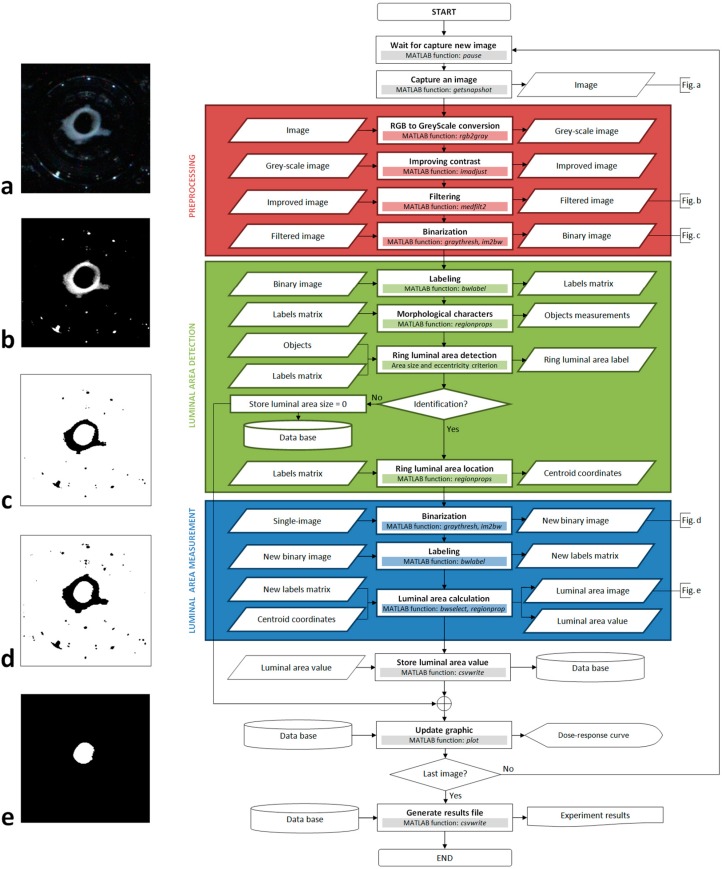
Image algorithm flowchart.

A new algorithm was developed to automatically detect and measure the luminal area of a blood vessel ring (and other luminal organs) from the images obtained. The algorithm is based on computer vision and photogrammetry techniques, which were implemented using the Image Acquisition [2] and Image Processing [3] toolboxes for MATLAB^®^. In a detailed data processing flowchart we highlight three main blocks: preprocessing, luminal area detection, and luminal area measurement (Figure 4). These three blocks are divided into different processes as described below.

### 3.1. Image Preprocessing

This first block in the algorithm includes four image enhancement processes: RGB to grey-scale conversion, contrast enhancement, filtering by median filter, and binarization. As the color of the image holds no information about the tissue’s response, we removed the RGB components to obtain a grey-scale image. The image’s histogram is modified to automatically enhance the contrast, so that the blood vessel ring stands out from the background image and other objects. To achieve this, the histogram is modified to give a grey values distribution similar to an inverted Gauss-Bell, in which the first threshold grey value is configured between 0% (black) and 50%, and the second threshold is between 75% and 100% (white). This step is very important because it reduces reflections, brightness and any undesirable bubbles.

After contrast enhancing, there are still some groups of pixels in the image that are too white, resulting from reflections and intense brightness due to the conditions of illumination. Many of these groups of pixels can be deleted through a binarization process if their intensity is attenuated. To achieve this we use a median filter that reduces the intensity of these pixels by calculating a new intensity value from neighboring pixels, without producing any blurring of the object’s edges (see the image obtained after applying these three adjustments in Figure 4b).

The binarization process reduces the grey-scale image (Figure 4b) to a binary image by applying a threshold routine (Figure 4c). We follow the Otsu method [4] included in the MATLAB^®^ libraries to set an appropriate threshold grey value for each image. The main advantage of this method is that the threshold value is calculated automatically and it is very efficient when the histogram is similar to an inverted Gauss-Bell distribution. Accordingly, the enhanced contrast and the filtering applied in the two stages indicated above allow the threshold value for the image to be optimized (see Figure 4c as an example of how the image appears after this process).

### 3.2. Detection of the Luminal Area

One of the key aspects of this algorithm is that it distinguishes the luminal area of the tissue from other objects. For this reason, we developed an efficient method to successfully identify and situate the lumen, again divided into four processes (Figure 4): labeling, calculation of the morphological features, identification of the luminal area, and location of the luminal area.

The labeling process takes the binary image from the previous stage as its input (Figure 4c). Basically, it consists in giving an identification number to each object present in the image, for which we have used the image-segmentation method outlined in [5] and that is included in MATLAB^®^ libraries. As a result of this process, we obtain a matrix of labels with the same dimensions as the input image. The value of each element corresponds to the label given to a pixel of the image in function of the object to which it belongs. However, one disadvantage of this process is that the larger the number of objects in the image the longer the computation time. For this reason, our algorithm searches for white objects enclosed by black pixels (for example, in Figure 4c there are only two white objects: the image background and the luminal area of the tissue, as opposed to more than twenty black objects).

The calculation of morphological features provides information about the shape of the objects. This information is taken from the matrix of labels using a specific MATLAB*^®^* function (see Figure 4) and from this, parameters such as the area, size, perimeter or eccentricity of objects can be calculated. These parameters allow us to differentiate some objects from others, and in our case, we have found that the best parameters to identify the luminal area are the size and eccentricity.

Since the area of the lumen is one of the largest objects in the image, we first removed all the objects that were smaller than a given pixel threshold. However, most objects that result from excessive brightness and reflections are difficult to remove and generally, they do not have a regular morphology. Conversely, the lumen of the rings is a very circular object and it stands out for its high level of eccentricity relative to the other objects. For this reason, after size filtering we choose the object with the highest eccentricity. After carrying out more than 3000 recognition tests, we found that this simple identification criterion has an error probability of approximately 0.001%. This process is the most critical step in the algorithm because the contractile response can only be measured once the tissue has been successfully identified. Once the lumen has been successfully identified, the algorithm uses the label associated with it to calculate the coordinates of the pixel closest to its centre from the matrix of labels obtained during labeling process.

### 3.3. Measurement of the Lumen Area

A priori, this last block seems redundant because we calculate the ring area in the previous step to distinguish it from other objects. However, this measurement was obtained from an image manipulated to facilitate the identification of the lumen area (Figure 4b) and the objects’ original appearance is considerably altered by binarization. In fact, when the appearance of the tissue is enhanced it becomes larger and more rounded, making it easier to distinguish to obtain its eccentricity value (Figure 4c). The problem is that the area measurement that we obtain is far from real and as such, the algorithm uses the original image (Figure 4a) along with the coordinates calculated in the previous block in order to obtain a measurement that is as accurate as possible.

To achieve this, the original image is binarized again without enhancement (generating an image such as that shown in Figure 4d), and this new binary image is labeled and a new matrix of labels is obtained. In this case, there are more objects because those that were removed from the image in the preprocessing block now appear again. However, as we now know the precise position of the lumen, it is easy to identify.

Subsequently, the luminal area of the ring is isolated into a binary image such as that shown in Figure 4e. This image is built from the new matrix of labels, where we only select those pixels whose label coincides with the label of the pixel located in the coordinates that we calculated in the previous block (centroid pixel). To shape the image, we paint the selected pixels white on a black background. Finally, the area is calculated (in pixels) by counting the number of white pixels that appears in the new binary image and this value is stored.

## 4. Experimental Section

We describe here an experiment carried out using our method to analyze the contractile effect of the α-adrenergic agonist phenylephrine on 15 rat aorta rings. This experiment was performed on male Sprague-Dawley rats that were anesthetized with diethyl ether, and sacrificed by cervical dislocation and exsanguination. The thoracic aorta was removed according to the procedure described in [6] and 25 aorta rings ≤1 mm thick were obtained under a stereoscopic microscope, extracting blood clots from the lumen. Each ring was placed in a small glass incubation chamber in 100 µL of Krebs-HEPES saline solution and maintained at 37 °C.

Before commencing the experiment the rings were allowed to stabilize for 40 min, changing the medium twice during this period. While the rings were stabilizing, the software application was prepared to set up the experimental parameters and adjust the video-camera lens in order to obtain a sharp image on the computer screen.

In the experiment, eight doses of phenylephrine were added at 8 min intervals in a cumulative manner. Specifically, the final concentration of the doses dispensed was 10 nM, 30 nM, 100 nM, 300 nM, 1 µM, 3 µM, 10 µM and 30 µM. To maintain the medium volume constant, each different dose was added in a 10 µL volume (a 10× concentration) after having previously removed 10 µL of the medium.

**Figure 5:**
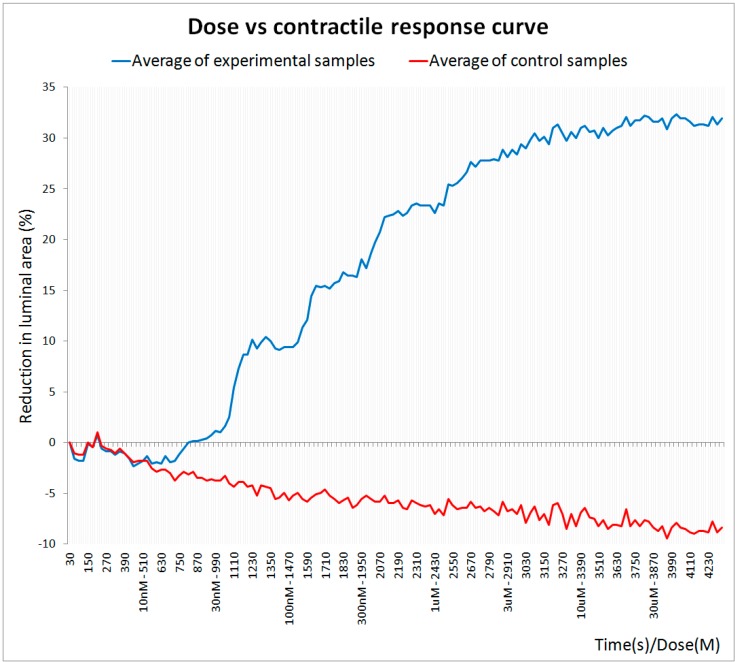
Dose-response curve obtained with the image processing algorithm.

The software application was configured to register the response of the rings at a rate of 2 frames/min. The application indicates when the next dose should be injected and the concentration that should be dispensed. As a result, the application generates a dose-response (contraction) curve. The program updates the plot after analyzing each new image, enabling the contractile effect to be observed on-line (see Figure 5 for the dose-response curve obtained in this experiment).

Each dot in the plot corresponds to the mean response of the 15 experimental (blue line) and 10 control (red line) rings. The curve is normalized to the average value of the initial lumen area, which corresponds to the baseline measurement. In this case, and due to the nature of the arterial tissue, it is impossible to obliterate the lumen and the maximum reduction in area produced by a vasoconstrictor like phenylephrine usually does not exceed 40%. This phenomenon is clearly evident in the dose-response curve (Figure 5), where a plateau is reached above a given concentration of phenylephrine at which the reduction in the lumen area remained around 30%.

## 5. Results and Discussion

We have implemented an innovative approach to measure the contraction of isolated blood vessels using an image sensor, obtaining results that are fully comparable to those obtained with mechanical transducers. For instance, the EC_50_ obtained for phenylephrine (169.5 ± 31.3 nM), as well as the displacement of the concentration-response curves to the right caused by the α_1_ antagonist prazosin, were in the nM range, as described using classical organ baths [7]. Using the Gaddum transformation [8], the K_i_ we obtained in this way for prazosin was 10 pM, which is similar to that found using a standard organ bath, both in our laboratory and elsewhere [7,9,10].

The use of this new approach offers certain advantages as it does not involve the use of any specific recording system (transducers, signal conditioning, or glassware: see Table 1). Moreover, as it avoids the use of the classic organ-bath, the amount of drugs, gases, buffers and solutions required is reduced. Likewise, since the rings are not tied to a sensor, rings with a thickness <1 mm can be used, minimizing tissue loss and facilitating multiple and simultaneous analyses. We initially thought that basal pretension would be necessary for recording, however, the aorta rings contracted and dilated (after contraction) in the same way as extension could be obtained with traditional systems. Histological analysis of arterial rings performed either immediately after isolation or after a 5 h experiment indicated that the arterial wall structures, including the endothelium, apparently suffered no damage. Moreover, the vasodilatation caused by acetylcholine on phenylephrine-precontracted arteries indicates the endothelium was still functional. We are currently adapting this technique to be used in 96-well plates. In addition, the system is also being successfully applied to other ring preparations like the rat trachea.

**Table 1:** Advantages over the classical method.

	Mechanical Sensor	Image Sensor
Average time to prepare and mount one tissue ring (*excluding sacrifice and dissection*)	20 s	2 s
Optimization of animal resources (*minimum ring thickness*)	4 mm	<1 mm
Optimization of drugs (*i.e.*, *for a n = 25 phenylephrine dose-response experiment*)	2.61 mg (*Minimum*)	0.041 mg (*Minimum*)
Gases	95% O_2_ + 5% CO_2_	Not Necessary
Buffers	6.25 L	0.05 L
Volume of isotonic solution per ring	4–50 mL	0.05–0.2 mL
Damage to tissue ring	Invasive (*The tissue is tied to the sensor by hooks*)	Non-invasive
Equipment	Mechanic sensor	Video-camera
	Transducer accessories	Telecentric lens *
	Signal conditioner	Illumination *
	Power supply	Computer
	Computer	

* Optional equipment.

## 6. Conclusions

A new and innovative method has been devised to measure the contractile effects of drugs on isolated blood vessels and other luminal organs based on an image sensor. This method is much cheaper than classical mechanical transducer based techniques and it can be implemented in any academic or industrial laboratory. Furthermore, it is very easy to use, it reduces the number of animals required, and it minimizes the drug and the solution volumes needed for each experiment.
